# Ranirestat Improved Nerve Conduction Velocities, Sensory Perception, and Intraepidermal Nerve Fiber Density in Rats with Overt Diabetic Polyneuropathy

**DOI:** 10.1155/2019/2756020

**Published:** 2019-11-18

**Authors:** Saeko Asano, Tatsuhito Himeno, Tomohide Hayami, Mikio Motegi, Rieko Inoue, Hiromi Nakai-Shimoda, Emiri Miura-Yura, Yoshiaki Morishita, Masaki Kondo, Shin Tsunekawa, Yoshiro Kato, Koichi Kato, Keiko Naruse, Jiro Nakamura, Hideki Kamiya

**Affiliations:** ^1^Division of Diabetes, Department of Internal Medicine, Aichi Medical University School of Medicine, Nagakute, Japan; ^2^Department of Medicine, Aichi Gakuin University School of Pharmacy, Nagoya, Japan; ^3^Department of Internal Medicine, Aichi Gakuin University School of Dentistry, Nagoya, Japan

## Abstract

Distal sensory-motor polyneuropathy is one of the most frequent diabetic complications. However, few therapies address the etiology of neurodegeneration in the peripheral nervous systems of diabetic patients. Several metabolic mechanisms have been proposed as etiologies of this polyneuropathy. In this study, we revisited one of those mechanisms, the polyol pathway, and investigated the curative effects of a novel strong aldose reductase inhibitor, ranirestat, in streptozotocin-induced diabetic rats with preexisting polyneuropathy. Twelve weeks after the onset of diabetes, rats which had an established polyneuropathy were treated once daily with a placebo, ranirestat, or epalrestat, over 6 weeks. Before and after the treatment, nerve conduction velocities and thermal perception threshold of hindlimbs were examined. After the treatment, intraepidermal fiber density was evaluated. As an ex vivo assay, murine dorsal root ganglion cells were dispersed and cultured with or without 1 *μ*mol/l ranirestat for 48 hours. After the culture, neurite outgrowth was quantified using immunological staining. Sensory nerve conduction velocity increased in diabetic rats treated with ranirestat (43.3 ± 3.6 m/s) compared with rats treated with placebo (39.8 ± 2.3). Motor nerve conduction velocity also increased in the ranirestat group (45.6 ± 3.9) compared with the placebo group (38.9 ± 3.5). The foot withdrawal latency to noxious heating was improved in the ranirestat group (17.7 ± 0.6 seconds) compared with the placebo group (20.6 ± 0.6). The decrease in the intraepidermal fiber density was significant in the diabetic placebo group (21.6 ± 1.7/mm) but not significant in the diabetic ranirestat group (26.2 ± 1.2) compared with the nondiabetic placebo group (30.3 ± 1.5). Neurite outgrowth was promoted in the neurons supplemented with ranirestat (control 1446 ± 147 *μ*m/neuron, ranirestat 2175 ± 149). Ranirestat improved the peripheral nervous dysfunctions in rats with advanced diabetic polyneuropathy. Ranirestat could have potential for regeneration in the peripheral nervous system of diabetic rats.

## 1. Introduction

Among several long-term complications in diabetic patients, diabetic polyneuropathy (DPN) is one of the most frequent complications and impairs the quality of life through sensorimotor dysfunction and amputation of lower limbs [1]. However, it is particularly noteworthy that a few therapies address the etiology of neurodegeneration in the peripheral nervous system (PNS) of diabetic patients. Currently, glucose-lowering therapy [2], alpha-lipoic acid [3], and aldose reductase inhibitor (ARI) epalrestat [4] are the available options to prevent the onset or progression of DPN. The pathogenesis of DPN has been vigorously investigated. In particular, mitochondrial oxidative stress and its dysfunction have been rigorously explored in experimental and clinical settings of type 2 diabetes [5–8]. However, it has been reported recently that mitochondrial uncoupling reagents have no effect on experimental DPN of rats with type 2 diabetes [9]. Given that the mitochondria hypothesis has almost been rejected, further insights into the pathogenesis of DPN should be provided.

Several metabolic mechanisms that are unbalanced in the diabetic milieu have been proposed as etiologies of DPN: polyol pathway, hexosamine pathway, glycations, and protein kinase C. As all of these metabolic imbalances evoke oxidative stress, many researchers have unsuccessfully focused on antioxidative therapies. However, as researchers of diabetic complications, we presently need to revisit those previously suggested metabolic mechanisms or identify other novel mechanisms. Hence, in this study, we scrutinized the effects of ranirestat, a novel ARI, which possesses higher inhibitory activity against aldose reductase (AR) than epalrestat, the only ARI clinically used for DPN [10]. AR, which is a key enzyme of the polyol pathway, is activated under hyperglycemia, generates polyols including sorbitol and fructose, and simultaneously induces NADH/NAD^+^ redox imbalance [11]. Given this etiological role of AR and polyol pathway in DPN, many ARIs have been discovered and tested. Despite several preclinical promising outcomes of ARIs, epalrestat has been exclusively approved as a commercial drug. More than a decade after the approval of epalrestat, a phase III study of the newly developing ARI ranirestat has finally been reported, in which ranirestat ameliorated nerve conduction velocities (NCVs) in diabetic patients with DPN [12]. However, as the phase III study failed to verify the significance of ranirestat on neuropathic symptoms and signs, the Pharmaceuticals and Medical Devices Agency, the regulatory authority in Japan, postponed the approval of the drug. Given that neuropathic symptoms and signs are not significant for evaluating the improvement of etiological mechanisms, we here attempted to increase the robustness of ranirestat utilizing pathological evaluation in DPN model rats. Matsumoto et al. have already proven the preventive effects of ranirestat in DPN employing morphological change of peripheral nerves in diabetic rats [13]. Thus, for the first time, we examined the curative effects of ranirestat in rats with preexisting DPN.

## 2. Research Design and Methods

### 2.1. Animals and Induction of Diabetes

Five-week-old male Wister rats (Japan SLC, Hamamatsu, Japan) were used. Diabetes was induced by intraperitoneal injection of streptozotocin (STZ) (50 mg/kg; Wako Pure Chemical, Osaka, Japan) dissolved in 0.1 mol/l citrate buffer at pH 4.5. Control nondiabetic rats received an equal volume of citric buffer. One week after STZ administration, the rats with plasma glucose concentrations of >16.7 mmol/l were selected as diabetic rats. Twelve weeks after the induction of diabetes, control nondiabetic and diabetic rats were treated once daily with ranirestat (Sumitomo Dainippon Pharma, Osaka, Japan) of 1.0 mg/kg body weight over 6 weeks. The ranirestat suspension was prepared in 0.5% aqueous tragacanth gum (Wako Pure Chemical) and administered with 2 ml/kg body weight of volume by gavage. Vehicle rats were administered the same amount of aqueous tragacanth gum without ranirestat (*n* = 8 − 10 in each group). Five rats in each group were treated with epalrestat (300 mg/kg body weight in aqueous tragacanth gum) (Sumitomo Dainippon Pharma) to compare the effects among ARIs. Before and after ranirestat treatment, casual blood glucose levels and body weights were examined. Blood glucose levels were measured by a FreeStyle Freedom™ (Nipro, Osaka, Japan). The Institutional Animal Care and Use Committee of Aichi Medical University approved the protocols of this experiment.

### 2.2. NCVs

Rats were maintained under anesthesia by inhalation of 1.5–3% isoflurane (Wako Pure Chemical) on an anesthetizer MK-AT210D (Muromachi Kikai, Tokyo, Japan) and placed on a heated pad in a room maintained at 25°C to ensure a constant rectal temperature of 37°C. Motor nerve conduction velocity (MNCV) was determined between the sciatic notch and ankle with Neuropak NEM-3102 instrument (Nihon-Kohden, Osaka, Japan), as previously described [14, 15]. The sensory nerve conduction velocity (SNCV) was measured between the knee and ankle with retrograde stimulation. The nerves were stimulated supramaximally by fine needle electrodes. The distance between these two sites were measured by a digital caliper and divided by the difference of take-off latency in the two sites.

### 2.3. Thermal Plantar Test

Before and after the treatments, hind paw withdrawal response to thermal stimuli of radiant heat was measured using a plantar test 7370 device (Ugo Basile, Gemonio, Italy). Using a Heat Flux Radiometer 37300 (Ugo Basile) to ensure the intensity, radiant heat was beamed onto the plantar surface of the hind paw. The paw withdrawal latencies were measured six times per session, separated by a minimum interval of 5 minutes. Paw withdrawals due to locomotion or weight shifting were not counted. Data are expressed as paw withdrawal latency in seconds.

### 2.4. Intraepidermal Nerve Fiber Density (IENFD)

After the 6-week treatment with ranirestat, epalrestat, or placebo, rats were sacrificed by an overdose of a combination anesthetic, which was prepared with 0.3 mg/kg of medetomidine, 4.0 mg/kg of midazolam, and 5.0 mg/kg of butorphanol. Plantar skin was excised and fixed for 5 hours in Zamboni's fixative (4% formaldehyde, 14% saturated picric acid, 0.1 M phosphate-buffered saline (PBS)) (Wako Pure Chemical) at 4°C. The fixed foot pads were frozen in O.C.T. Compound (Sakura Finetechnical, Tokyo, Japan) after cryoprotection by sequential incubation in 10, 20, and 30% sucrose (Wako Pure Chemical) for 6-12 hours at 4°C.

For immunohistochemistry, skin tissue was sectioned into 30 *μ*m of thickness using a cryostat CM 3050S (Leica Microsystems, Wetzlar, Germany). After 60 s microwave irradiation in citrate buffer (pH 6.0), the sections were treated with 0.3% Triton X-100 (Wako Pure Chemical) solution in PBS for 10 minutes and then blocked with 1% bovine serum albumin (Wako Pure Chemical) for 60 minutes at 4°C. Rabbit polyclonal antibody against ubiquitin C-terminal hydrolase 1, also called protein gene product 9.5 (PGP9.5) (1 : 500; RPCA-UCHL1, EnCor Biotechnology Inc., Gainesville, FL, United States of America), was applied to the sections at 4°C overnight. After rinsing with PBS three times, Alexa Fluor 488-coupled goat anti-rabbit IgG antibody (1 : 200; Life Technologies, Carlsbad, CA, United States of America) was loaded for 1 hour at room temperature. Sections were counterstained with 4,6-diamidino-2-phenylindole (DAPI) (Merck, Darmstadt, Germany). Images were captured using a confocal laser scanning microscope LSM710 (Zeiss, Oberkochen, Germany) and analyzed using Zen software (Zeiss).

Nerve fibers stained with anti-PGP9.5 antibody were counted as previously reported [16]. In brief, each individual nerve fiber with branching inside the epidermis was counted as one; a nerve fiber with branching in the dermis was counted separately. Three fields from each section were randomly selected for the quantification of IENFDs. Nerve fibers distributed within 20 *μ*m of thickness in each section were counted. IENFDs were derived and expressed as epidermal nerve fiber numbers per length of the epidermal basement membrane (fibers/mm).

### 2.5. Primary Culture of Dorsal Root Ganglion (DRG) Neurons and Evaluation of Neurite Outgrowth

DRG neuron cultures were prepared from 5-week-old male C57BL/6 mice (Japan SLC) and 3-week-old Sprague Dawley rats (Japan SLC) as previously described [17]. In brief, DRGs were collected, dissociated by collagenase (Wako Pure Chemical) and diluted in a medium consisting of Dulbecco's Modified Eagle Medium/Nutrient Mixture F-12 media (Thermo Fisher Scientific, Waltham, MA, United States of America), 17.5 mM glucose, and 30 nM selenium (Thermo Fisher Scientific). Isolated DRG neurons were seeded on glass coverslips coated with poly-L-lysine (Thermo Fisher Scientific). DRG neurons were cultured with or without 1 *μ*mol/l ranirestat (Sumitomo Dainippon Pharma).

After 24 hours of culture, the cells were fixed with 4% (wt/vol) paraformaldehyde for 20 minutes and blocked with 1% bovine serum albumin (Wako Pure Chemical) for 30 minutes at 4°C. Thereafter, the DRG neurons were immunologically stained with rabbit polyclonal antineurofilament heavy-chain antibody (1 : 500; Merck) and visualized with Alexa Fluor 488-coupled goat anti-rabbit IgG antibody (1 : 300; Life Technologies). Images were captured utilizing fluorescence microscopy Olympus IX73 (Olympus, Tokyo, Japan). Neurite outgrowth was observed in 10-20 neurons per coverslip and evaluated by ImageJ software (National Institutes of Health, Bethesda, MD, United States of America).

## 3. Results

### 3.1. Hyperglycemia and Body Weight during the Treatment with Ranirestat

At 12 weeks after the STZ administration, diabetic rats (DM) showed severe hyperglycemia (*p* < 0.05) and significantly reduced body weight gain (*p* < 0.05) compared with nondiabetic rats (non-DM) (Table 1). Ranirestat or epalrestat treatment for 6 weeks induced no significant difference in body weight or blood glucose levels in any group.

### 3.2. Ranirestat Improved Delayed MNCV in Diabetic Rats

**Figure 1:**
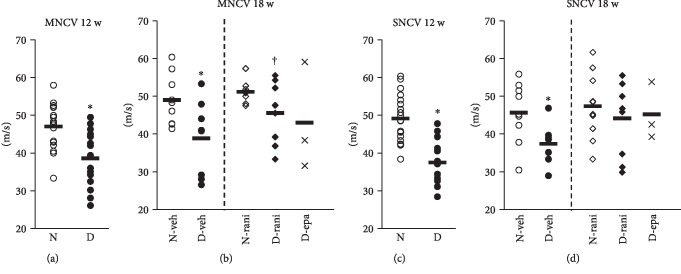
Amelioration of nerve conduction velocities in hindlimbs of diabetic rats treated with ranirestat. Nerve conduction velocities decreased in the diabetic rats 12 weeks after induction of diabetes (a, c). The decrease was diminished in the diabetic rats after 6-week treatment with ranirestat (b, d). MNCV: motor nerve conduction velocity; SNCV: sensory nerve conduction velocity; 12 w: 12 weeks after induction of diabetes; 18 w: 18 weeks after induction of diabetes; N: nondiabetic rats; D: diabetic rats; N-veh: nondiabetic rats treated with placebo; D-veh: diabetic rats treated with placebo; N-rani: nondiabetic rats treated with ranirestat; D-rani: diabetic rats treated with ranirestat; D-epa: diabetic rats treated with epalrestat. ^∗^*p* < 0.05 compared with N or N-veh and ^†^*p* < 0.05 compared with D-veh. *n* = 15~18 at 12 w, *n* = 8~10 in groups treated with placebo or ranirestat, and *n* = 3 in D-epa. Bold horizontal line in each group demonstrates an average value.

MNCV and SNCV of diabetic rats were significantly delayed compared with those of normal rats (MNCV: DM 38.6 ± 1.9 m/s, non-DM 47.1 ± 1.4, *p* = 0.0010; SNCV: non-DM 49.1 ± 1.5, DM 39.8 ± 2.3, *p* = 0.0013) (Figures 1(a) and 1(b)). The delay in MNCV and SNCV of DM was significantly restored after 6-week administration of ranirestat (MNCV: vehicle 38.9 ± 3.5, ranirestat 45.6 ± 3.0, *p* = 0.0448; SNCV: vehicle 39.6 ± 2.9, ranirestat 43.4 ± 3.6, *p* = 0.0620). The administration of ranirestat caused no significant change of NCVs in non-DM (Figures 1(c) and 1(d)).

### 3.3. Treatment with Ranirestat Decreased Withdrawal Latency from Noxious Heat to Planta Pedis in Diabetic Rats

**Figure 2:**
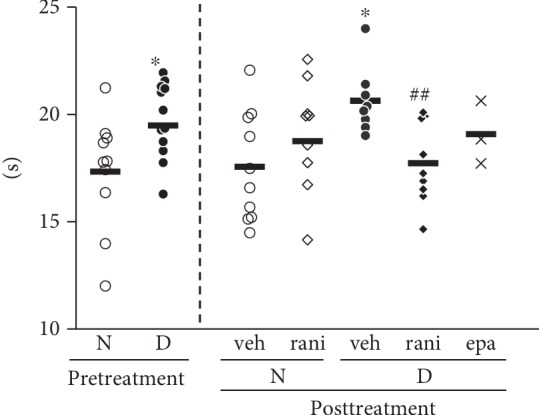
Amelioration of thermal perception threshold in planta pedis of diabetic rats treated with ranirestat. Thermal perception threshold increased in the diabetic rats 12 weeks after induction of diabetes. The increase was diminished in the diabetic rats after 6-week treatment with ranirestat. N: nondiabetic rats; D: diabetic rats; veh: rats treated with placebo; rani: rats treated with ranirestat; epa: rats treated with epalrestat. ^∗^*p* < 0.05 compared with N or nondiabetic vehicle and ^##^*p* < 0.005 compared with diabetic vehicle. *n* = 15~18 at 12 w, *n* = 8~10 in groups treated with placebo or ranirestat, and *n* = 3 in rats treated with epalrestat. Bold horizontal line in each group demonstrates an average value.

The foot withdrawal latency to noxious radiant heating was significantly prolonged in DM compared with non-DM after 12-week duration (non-DM 17.3 ± 0.8 seconds, DM 19.5 ± 0.5, *p* = 0.0353) (Figure 2). After the 6-week treatment, the latency in DM treated with ranirestat was ameliorated compared with DM treated with placebo (vehicle 20.6 ± 0.6, ranirestat 17.7 ± 0.6, *p* = 0.0039). The treatment in non-DM caused no significant alternation of the latency (vehicle 17.5 ± 0.8, ranirestat 18.7 ± 0.8, *p* = 0.3162).

### 3.4. IENFDs Were Higher in DM Treated with Ranirestat Compared with the Vehicle DM

**Figure 3:**
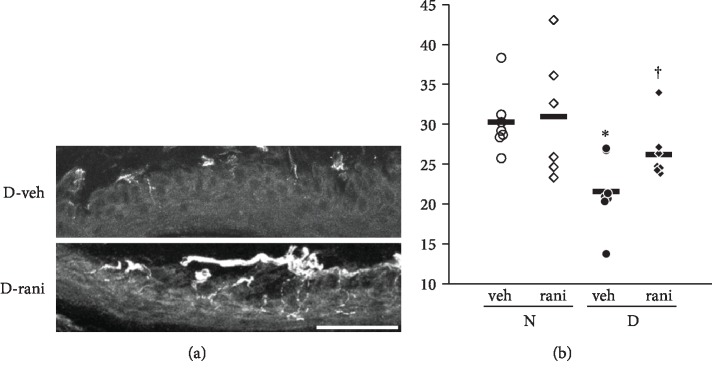
Intraepidermal nerve fiber density (IENFD) after the treatment with ranirestat. (a) Representative photos of IENFs in diabetic rats treated with or without ranirestat. (b) Quantification of the IENFD revealed a significant decrease in untreated diabetic rats and the significant amelioration by ranirestat treatment. N: nondiabetic rats; D: diabetic rats; veh: rats treated with placebo; rani: rats treated with ranirestat. ^∗^*p* < 0.05 versus N treated with placebo, ^†^*p* < 0.05 versus D treated with ranirestat, and *n* = 6–8 in each group. Scale bar: 50 *μ*m.

The IENFDs of foot pads in DM significantly decreased compared with non-DM after 18 weeks of diabetes (non-DM 30.3 ± 1.5/mm, DM 21.6 ± 1.7, *p* = 0.0022) (Figure 3). However, the decrease was not evident in DM treated with ranirestat (DM treated with ranirestat 26.2 ± 1.2, *p* = 0.0384 versus DM treated with placebo). The administration in non-DM provided no significant difference of the densities (non-DM treated with ranirestat 30.9 ± 3.2, *p* = 0.8496 versus non-DM treated with placebo).

### 3.5. Neurite Outgrowth Was Promoted in the Primary Culture of DRG Neurons Supplemented with Ranirestat

**Figure 4:**
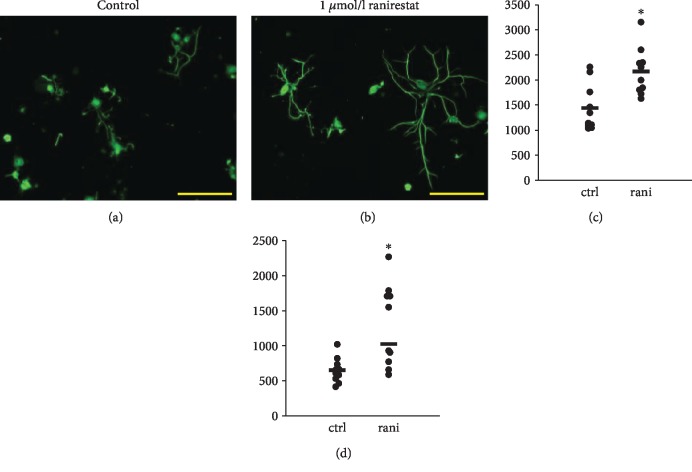
Neurite outgrowth of dorsal rood ganglion (DRG) neurons was elongated by ranirestat. Ranirestat (1 *μ*mol/l) increased total neurite length in each neuron. (a) Representative figure of murine DRG neurons cultured with control medium. (b) Representative figure of mouse DRG neurons cultured with 1 *μ*g/l ranirestat. Scale bar: 200 *μ*m. (c) Quantified data of neurite outgrowth in mouse DRG culture. (d) Quantified data of neurite outgrowth in rat DRG culture. ctrl: control medium; rani: medium supplemented with 1 *μ*mol/l ranirestat. ^∗^*p* < 0.05 versus ctrl. *n* = 10 in each group.

In the primary culture of DRG, immunostaining with antineurofilament H antibody visualized large neurons. In those neurons, 48-hour exposure to ranirestat promoted neurite elongation (mouse: control 1446 ± 147 *μ*m/neuron, ranirestat 2175 ± 149, *p* = 0.0026; rat: control 654 ± 177, ranirestat 1292 ± 582, *p* = 0.0039) (Figure 4).

## 4. Discussion

In the current study, we revealed the beneficial effects of ranirestat in type 1 diabetes model rats, which expressed observable impairments of sensory perception and slowed NCVs. Epalrestat, the only clinically available ARI, produces better effects in the early stage than in the advanced stage of DPN. Therefore, it is worthwhile to reveal the effects of the novel ARI ranirestat in the advanced stage of DPN. The treatment with ranirestat ameliorated perception and NCVs. Furthermore, the treatment provided a pathological benefit in IENFDs. Thus, as a result, we suggested an additional advantage of ARI in DPN.

To understand the pathogenesis of DPN, researchers have suggested many hypotheses. In particular, the hypothesis of mitochondrial oxidative stress was the most theorized and supported mechanism. However, Hinder et al. recently clarified that no favorable outcome was derived from a direct intervention for mitochondrial oxidative dysfunction in DPN and other diabetic complications [9]. Researchers of diabetic complications, in consequence, should scrutinize other old and new hypotheses.

We are focusing on the polyol pathway as one of the most important potential pathogeneses of DPN. The effectiveness of epalrestat on DPN has been verified in early-stage patients. However, the effects on nervous dysfunction were limited in late-stage DPN patients [4]. Compared with epalrestat, ranirestat has a stronger inhibitory activity against aldose reductase and significantly reduced the accumulation of sorbitol and fructose in sciatic nerves of diabetic rats [18]. In the phase III trial in Japan, ranirestat increased NCVs in DPN patients after a 52-week treatment [12]. However, as clinical symptoms evaluated with modified Toronto Clinical Neuropathy Score were not significantly improved by treatment with ranirestat, the regulatory authority postponed the approval of the drug. We believe that the current study will strengthen the evidence for the advantages of ranirestat and support its clinical application.

Unlike previous studies in which ranirestat was administered soon after the induction of diabetes in rats [13], we started the treatment with ranirestat after confirmation of sensory perception dysfunction and decreased NCVs in diabetic rats. Therefore, the outcomes in the current study successfully provided the effect of ranirestat in late preventive intervention to DPN.

In addition, this is the first study that revealed a significant difference in neuropathological finding, i.e., IENFD. IENFD is widely used as a surrogate marker of small nerve fiber damage in diabetic patients [19]. Because it is difficult in a short time to retrieve morphological changes in large fibers of diabetic rodents [20], previous papers discovered only subtle morphological differences in myelinated fibers of diabetic rats treated with ranirestat [13]. To compensate, we examined small fiber damage in rats exposed to longer-term hyperglycemia.

As IENFD is a pathological consequence, it is difficult to distinguish whether treatments contributed to neurodegeneration or neuroregeneration. Thus, we alternatively utilized the *ex vivo* experiment, i.e., neurite outgrowth of DRG neurons to evaluate the neuroregenerative effects of ranirestat. The ranirestat supplementation in the primary DRG culture promoted neurite outgrowths. Although the promotion of neurite outgrowth might indicate the neuroregenerative effect, the intracellular molecular mechanism remains to be clarified. However, a recent report may explain the mechanism. In that report, the aldose reductase inhibitor sorbinil promoted neurite outgrowth through activation of SIRT2 and its downstream components including AMP-activated protein kinase and peroxisome proliferator-activated receptor *γ* coactivator 1-*α* [21]. We plan to investigate the contribution of the SIRT2 pathway in a future study.

The current study could demonstrate that the novel ARI have a potential to reverse neurodysfunction in DPN. However, there are several limitations to this study. First, although we planned to compare the effectiveness between the novel ARI ranirestat to the clinically available ARI epalrestat, we unfortunately failed to examine the rats treated with epalrestat because of their high mortality during the long exposure to hyperglycemia. However, MNCV and thermal perception in rats treated with epalrestat showed a trend of inferior outcomes compared with those in rats treated with ranirestat and a trend of superior outcomes compared with those in the vehicle diabetic rats. Second, the nondiabetic rats had higher blood glucose levels after an 18-week intervention compared with the levels at the baseline. The increase in casual blood glucose levels may be caused by aging, isoflurane anesthesia [22], and stress by animal handling [17]. Nevertheless, as the higher blood levels were not significantly different between rats treated with placebo and ranirestat, the interpretation of the current study will not be impacted by the increase. Third, the most validated examination of IENFD was evaluated only at the endpoint. Because it is difficult to repeat skin biopsies in planta pedis of rats, corneal confocal microscopy may become a promising alternative to evaluate small fiber impairments in animal DPN models [23]. Fourth, it is difficult to interpret the result of *ex vivo* neurite outgrowth. The elongation of neurites is traditionally interpreted as a preferable effect for neuroregeneration. However, vast reagents, the neuroregenerative activities of which had been verified using *ex vivo* experiments, failed to replicate the beneficial effects in clinical settings [24, 25]. Therefore, we, in the future, would like to justify the neuroregenerative ability through exploring other cellular aspects, e.g., proliferation, cytotoxicity, apoptosis, and neuroelectric response.

## 5. Conclusions

In conclusion, ranirestat could have potential for regeneration in the peripheral nervous system of diabetic rats. Therefore, further clinical trials are warranted.

## Figures and Tables

**Table 1:** Body weight and blood glucose levels in nondiabetic and diabetic rats.

	Nondiabetic rats			Diabetic rats		
	Pre-Tx	Post-Tx		Pre-Tx	Post-Tx	
		Placebo	Rani		Placebo	Rani
Number	19-20	4-6	8	16	5	7
CBG (mM)	6.7 ± 1.3	16.8 ± 4.6^†^	13.2 ± 2.9^†^	28.3 ± 3.9^∗^	29.6 ± 4.9^∗^	30.5 ± 4.1^∗^
BW (g)	518 ± 32	566 ± 35	485 ± 96	254 ± 54^∗^	241 ± 34^∗^	253 ± 67^∗^

Results are means ± SD. Tx: treatment; Rani: ranirestat; CBG: casual blood glucose; BW: body weight. ^∗^*p* < 0.05 versus pre-Tx nondiabetic rats and ^†^*p* < 0.05 versus pre-Tx nondiabetic rats.

## Data Availability

The data used to support the findings of this study are available from the corresponding author upon request.

## References

[1] Kobayashi M., Zochodne D. W. (2018). Diabetic neuropathy and the sensory neuron: new aspects of pathogenesis and their treatment implications. *Journal of Diabetes Investigation*.

[2] Lachin J. M., Bebu I., Bergenstal R. M. (2017). Association of glycemic variability in type 1 diabetes with progression of microvascular outcomes in the diabetes control and complications trial. *Diabetes Care*.

[3] Ziegler D., Low P. A., Freeman R., Tritschler H., Vinik A. I. (2016). Predictors of improvement and progression of diabetic polyneuropathy following treatment with *α*-lipoic acid for 4 years in the NATHAN 1 trial. *Journal of Diabetes and its Complications*.

[4] Hotta N., Kawamori R., Fukuda M., Shigeta Y., The Aldose Reductase Inhibitor-Diabetes Complications Trial Study Group (2012). Long‐term clinical effects of epalrestat, an aldose reductase inhibitor, on progression of diabetic neuropathy and other microvascular complications: multivariate epidemiological analysis based on patient background factors and severity of diabetic neuropathy. *Diabetic Medicine*.

[5] Ziegler D., Low P. A., Litchy W. J. (2011). Efficacy and safety of antioxidant treatment with *α*-lipoic acid over 4 years in diabetic polyneuropathy: the NATHAN 1 trial. *Diabetes Care*.

[6] Strom A., for the GDS Group, Kaul K. (2017). Lower serum extracellular superoxide dismutase levels are associated with polyneuropathy in recent-onset diabetes. *Experimental & Molecular Medicine*.

[7] Ziegler D., Buchholz S., Sohr C., Nourooz-Zadeh J., Roden M. (2015). Oxidative stress predicts progression of peripheral and cardiac autonomic nerve dysfunction over 6 years in diabetic patients. *Acta Diabetologica*.

[8] Sifuentes-Franco S., Pacheco-Moises F. P., Rodriguez-Carrizalez A. D., Miranda-Diaz A. G. (2017). The role of oxidative stress, mitochondrial function, and autophagy in diabetic polyneuropathy. *Journal Diabetes Research*.

[9] Hinder L. M., Sas K. M., O'Brien P. D. (2019). Mitochondrial uncoupling has no effect on microvascular complications in type 2 diabetes. *Scientific Reports*.

[10] Ishibashi Y., Matsui T., Matsumoto T., Kato H., Yamagishi S. (2016). Ranirestat has a stronger inhibitory activity on aldose reductase and suppresses inflammatory reactions in high glucose-exposed endothelial cells. *Diabetes & Vascular Disease Research*.

[11] Yan L. J. (2018). Redox imbalance stress in diabetes mellitus: role of the polyol pathway. *Animal Model Exp Med.*.

[12] Sekiguchi K., Kohara N., Baba M. (2019). Aldose reductase inhibitor ranirestat significantly improves nerve conduction velocity in diabetic polyneuropathy: A randomized double‐blind placebo‐controlled study in Japan. *Journal of Diabetes Investigation*.

[13] Matsumoto T., Ono Y., Kuromiya A., Toyosawa K., Ueda Y., Bril V. (2008). Long-term treatment with ranirestat (AS-3201), a potent aldose reductase inhibitor, suppresses diabetic neuropathy and cataract formation in rats. *Journal of Pharmacological Sciences*.

[14] Kondo M., Kamiya H., Himeno T. (2015). Therapeutic efficacy of bone marrow‐derived mononuclear cells in diabetic polyneuropathy is impaired with aging or diabetes. *J Diabetes Investig.*.

[15] Omi M., Hata M., Nakamura N. (2017). Transplantation of dental pulp stem cells improves long-term diabetic polyneuropathy together with improvement of nerve morphometrical evaluation. *Stem Cell Research & Therapy*.

[16] Beiswenger K. K., Calcutt N. A., Mizisin A. P. (2008). Epidermal nerve fiber quantification in the assessment of diabetic neuropathy. *Acta Histochemica*.

[17] Ghosal S., Nunley A., Mahbod P. (2015). Mouse handling limits the impact of stress on metabolic endpoints. *Physiology & Behavior*.

[18] Ota A., Kakehashi A., Toyoda F. (2013). Effects of long-term treatment with ranirestat, a potent aldose reductase inhibitor, on diabetic cataract and neuropathy in spontaneously diabetic torii rats. *Journal Diabetes Research*.

[19] Pittenger G. L., Ray M., Burcus N. I., McNulty P., Basta B., Vinik A. I. (2004). Intraepidermal nerve fibers are indicators of small-fiber neuropathy in both diabetic and nondiabetic patients. *Diabetes Care*.

[20] Kamiya H., Zhang W., Ekberg K., Wahren J., Sima A. A. (2006). C-peptide reverses nociceptive neuropathy in type 1 diabetes. *Diabetes*.

[21] Schartner E., Sabbir M. G., Saleh A. (2018). High glucose concentration suppresses a SIRT2 regulated pathway that enhances neurite outgrowth in cultured adult sensory neurons. *Experimental Neurology*.

[22] Tanaka K., Kawano T., Tomino T. (2009). Mechanisms of impaired glucose tolerance and insulin secretion during isoflurane anesthesia. *Anesthesiology*.

[23] Davidson E. P., Coppey L. J., Shevalye H., Obrosov A., Yorek M. A. (2018). Vascular and neural complications in type 2 diabetic rats: improvement by sacubitril/valsartan greater than valsartan alone. *Diabetes*.

[24] Ishima T., Nishimura T., Iyo M., Hashimoto K. (2008). Potentiation of nerve growth factor-induced neurite outgrowth in PC12 cells by donepezil: role of sigma-1 receptors and IP_3_ receptors. *Progress in Neuro-Psychopharmacology & Biological Psychiatry*.

[25] Page M., Pacico N., Ourtioualous S., Deprez T., Koshibu K. (2015). Procognitive compounds promote neurite outgrowth. *Pharmacology*.

